# Intraspecific Competition Affects the Pupation Behavior of Spotted-Wing Drosophila (*Drosophila suzukii*)

**DOI:** 10.1038/s41598-019-44248-6

**Published:** 2019-05-23

**Authors:** Cherre Sade Bezerra Da Silva, Kyoo R. Park, Rachel A. Blood, Vaughn M. Walton

**Affiliations:** 1 Department of Horticulture, Oregon State University, 4017 Agricultural and Life Sciences Building, Corvallis, OR 97331 USA; 2 Present Address: Embrapa Algodão, Rua Oswaldo Cruz 1143, Campina Grande, PB 58428-095 Brazil

**Keywords:** Behavioural ecology, Entomology

## Abstract

In *Drosophila*, intraspecific competition (IC) may cause stress, cannibalism, and affect survival and reproduction. By migrating to less crowded environments, individuals can escape IC. Larvae of spotted-wing drosophila (SWD, *Drosophila suzukii*) are often exposed to IC. They are known to pupate either attached to or detached from their hosts. Here, we hypothesized that SWD pupates detached from the larval host as a means to escape IC and increase their survival and fitness. Under laboratory conditions, IC resulted in increased pupation detached from the larval host in both cornmeal medium and blueberry fruit. Males were more prone to detached pupation than females. In blueberry, IC-exposed larvae pupated farther away from the fruit relative to singly-developed individuals. Detached pupation was associated to survival and fitness gains. For example, larvae that displayed detached pupation showed shorter egg-pupa development times, higher pupa-adult survival, and larger adult size relative to fruit-attached individuals. These findings demonstrate that SWD larvae select pupation sites based on IC, and that such a strategy is associated with improved survival and fitness. This information contributes to a better understanding of SWD basic biology and behavior, offering insights to the development of improved practices to manage this pest in the field.

## Introduction

Spotted-wing drosophila (SWD), *Drosophila suzukii* (Matsumura), is a species of vinegar fly native to Southeast Asia^1–3^. It was detected in the mainland United States (California) and in Europe (Spain) for the first time in 2008, and has since been found in 41 US states and 18 European countries^4,5^. As an exception rather than a rule within its genus, SWD actively seeks and successfully attacks healthy (undamaged) fruits, characteristics that have been attributed to its specialized olfactory system and serrated ovipositor, respectively^6^. These features, along with a high reproductive capacity, short life-cycle^7^, flexibility to persist in diverse climates and weather conditions, polyphagy, limited effective natural enemies, among other characteristics, have contributed to the status of SWD as a key pest of berries and stone fruits in many invaded areas^3,4,8–11^.

Currently, chemical control has formed the foundation of tactics adopted by growers to manage SWD, with organophosphate, pyrethroid, and spinosyn among the most effective classes of insecticides^12–14^. However, fruit growers want to reduce reliance on insecticides to be environmentally sustainable. Knowledge about the species’ ecology, behavior, and evolution may give insights into the development of more sustainable management tools^6,15,16^. In *Drosophila*, pupation behavior can be affected by strain, temperature, humidity, texture of the substrate, and the presence of heterospecific competitors^17–19^. Specifically in *D. melanogaster*, the locomotor activity of larvae affects the rates at which they are attacked by parasitoids^20^. Mature larvae will select microenvironments that will enhance pupal survival, a component of fitness^18^.

The ability of SWD to pupate either attached to or detached from its host fruit has been known for decades^21^, and larvae have been reported exiting from hanging and fallen fruit to pupate in the soil in the field^22^. Research on biological control has focused on SWD larvae/pupae found in soil by means of entomopathogenic fungi, nematodes, and predators^23–26^. Hence, understanding the factors that lead SWD larvae to select one pupation site over another, as well as the consequences of such choices, will contribute to the knowledge of the population dynamics, and give insights to the development of better management tools for this important agricultural pest.

In laboratory, SWD pupation usually occurs within the host (a Petri dish containing standard cornmeal medium) (Supplementary Fig. S1a, left). Sometimes pupae can also be found on the lid, walls, and bottom of the culture boxes, even when patches of cornmeal with no pupae are available within the host (Supplementary Fig. S1a, right). When comparing both situations, we noticed that containers with scattered pupation (latter case) contained higher densities of individuals compared to containers with concentrated pupation within the rearing media. This observation suggested the possibility that intraspecific competition (IC) affects the selection of pupation sites by SWD larvae. It is known that gravid SWD females often lay more than one egg per fruit^27,28^, thus exposing their offspring to IC, while potentially increasing development time and reducing survival^29^. To date, the impact of IC on SWD larval behavior and subsequent survival and developmental benefits is however unknown.

The goal of this work was to evaluate the effect of IC on the pupation behavior of SWD larvae. We hypothesized that (1) IC results in detached (*off*-host) pupation as opposed to attached (*in*- or *on*-host) pupation; and (2) increased survival and fitness are associated with this behavior. Our results have clearly shown that solitary SWD larvae as well as those under low levels of IC prefer to pupate attached to their host. However, as the number of individuals per host increases (i.e., as IC arises), that initial preference is linearly switched to detached pupation. Importantly, detached individuals had shorter development, higher survival, and larger size, among other benefits, than the attached ones, leading us to conclude that detached pupation is associated with survival and fitness gains.

## Results

**Figure 1:**
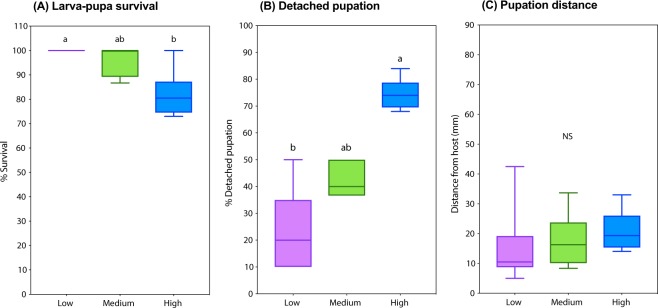
Response of spotted-wing drosophila (*Drosophila suzukii*) to low, medium, and high larval densities (respectively 10, 30, and 100 3^rd^ instar larvae per assay tube containing 3 g of artificial diet) in cornmeal medium. (**A**) Percentage of larva-pupa survival (Kruskal-Wallis *H* = 10.18, *P* = 0.0019; Dunn’s *P* = 0.0063). (**B**) Percentage of larvae that displayed pupation detached from the host as opposed to pupation attached to the host (*H* = 12.56, *P* < 0.0001; Dunn’s *P* = 0.0017). (**C**) Distance (mm) between detached pupae and host (*H* = 3.594, *P* = 0.1682). *N* = 5–6.

In tubes containing artificial diet (cornmeal medium), we found that increasing larval density significantly decreased SWD’s larva-pupa survival (Kruskal-Wallis *H* = 10.18, *P* = 0.0019; Dunn’s *P* = 0.0063, Fig. 1A) and increased detached pupation (*H* = 12.56, *P* < 0.0001; Dunn’s *P* = 0.0017, Fig. 1B, Supplementary Fig. S1b). Larval density did not affect distance between pupa and host (*H* = 3.594, *P* = 0.1682, Fig. 1C).

**Figure 2:**
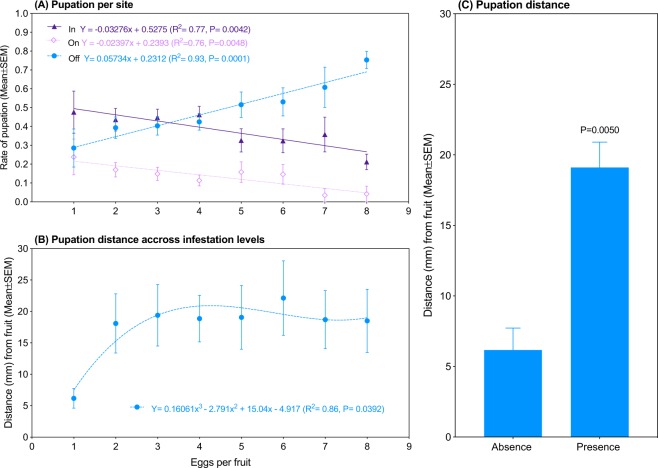
Response of spotted-wing drosophila (*Drosophila suzukii*) to egg density in blueberry fruit. (**A**) Rates of pupation *in*, *on*, and *off* a blueberry fruit, and (**B**) distance (mm) between pupae and fruit. (**C**) Pupation distance of individuals that developed under absence of IC vs. under its presence (Two-tailed Mann-Whitney U-test, U = 128, P = 0.0050). *N* = 19–37.

In tubes containing natural diet (1 blueberry fruit) infested with 1, 2, 3, 4, 5, 6, 7, or 8 SWD eggs, we initially investigated the rates specifically of *in-*, *on-*, and *off*-fruit pupations as opposed to attached and detached pupation as in the previous experiment. This was done because in blueberry, the fruit's skin makes it much easier to distinguish *in*- and *on*-fruit pupations from one another (i.e., on = attached to the exterior surface of the skin, in=attached to the skin's internal surface and/or to fruit’s flesh), whereas in cornmeal medium the lack of skin makes that separation much harder. No significant differences were found among those three pupation sites at the density of 1 egg per fruit (*H* = 2.952, *P* = 0.2285, Fig. 2A, Supplementary Fig. S2). However, increasing density consistently elevated *off*-fruit pupation from 28.6% at 1 egg per fruit, to 75.3% (2.7-fold higher) at 8 eggs per fruit (*F*_*1,6*_ = 80.83, *P* = 0.0001, Fig. 2A). Additionally, both *in*– and *on*–fruit pupation were decreased as egg density increased (*in*: *F*_*1,6*_ = 20.02, *P* = 0.0042; *on*: *F*_*1,6*_ = 18.96, *P* = 0.0048, Fig. 2A). Since both *in*- and *on*-fruit pupations had negative slopes and referred to individuals that were attached to the fruit, for the following analyses they were grouped and referred to as “attached pupation”.

### Pupation distance

The distance between the detached pupa and the blueberry fruit, i.e., the pupation distance, was consistently extended from 6.2 mm at 1 egg per fruit (absence of IC) to *ca*. 19 mm at 2–8 eggs per fruit (presence of IC) (*F*_*3,4*_ = 7.64, *P* = 0.0392, R^2^ = 0.86, Fig. 2B). Mann-Whitney *U* test confirmed that such distance was shorter under absence of IC than under its presence (*U* = 128, *P* = 0.0050, Fig. 2C).

### Survival

**Figure 3:**
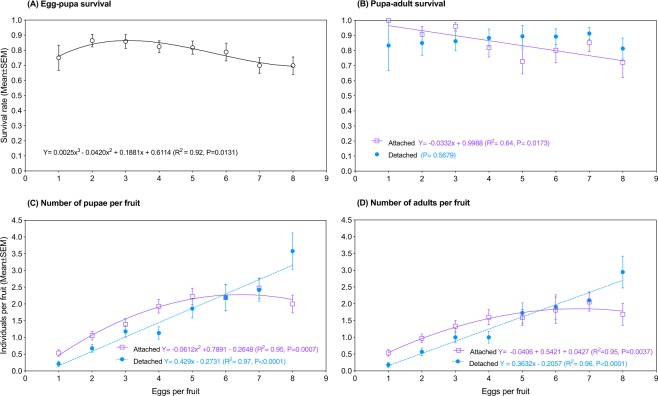
Response of spotted-wing drosophila (*Drosophila suzukii*) to egg density in blueberry fruit when individuals displayed pupation attached to and detached from the fruit. (**A**) Egg-pupa survival. (**B**) Pupa-adult survival. (**C**) Number of pupae per fruit. (**D**) Number of adults per fruit. Curves not shown when P > 0.05. *N* = 19–37.

Egg density caused a cubic response in the egg-pupa survival (pupae/eggs) of SWD, with a peak of 0.9 pupae/egg at the density of 2 eggs per fruit (*F*_*3,4*_ = 9.47, *P* = 0.0132, R^2^ = 0.92, Fig. 3A). No effect of egg density was found on the pupa-adult survival (adults/pupae) of individuals that displayed detached pupation (*F*_*1,6*_ = 0.365, *P* = 0.5679). Conversely, in individuals that pupated attached to the fruit, pupa-adult survival decreased linearly as egg density rose (*F*_*1,6*_ = 10.6, *P* = 0.0173, R^2^ = 0.64, Fig. 3B). The number of SWD individuals that reached the stage of pupa per blueberry fruit responded either linearly or quadratically to egg density, depending on if they pupated detached from or attached to the fruit, respectively (detached: *F*_*1,6*_ = 110.5, *P* < 0.0001, R^2^ = 0.95; attached: *F*_*3,4*_ = 63.82, *P* = 0.0008, R^2^ = 0.97, Fig. 3C). Similar patterns were observed for the number of SWD adults emerged per blueberry fruit (detached: *F*_*1,6*_ = 158, *P* < 0.0001, R^2^ = 0.96; attached: *F*_*3,4*_ = 28.55, *P* = 0.0037, R^2^ = 0.95, Fig. 3D).

### Development

**Figure 4:**
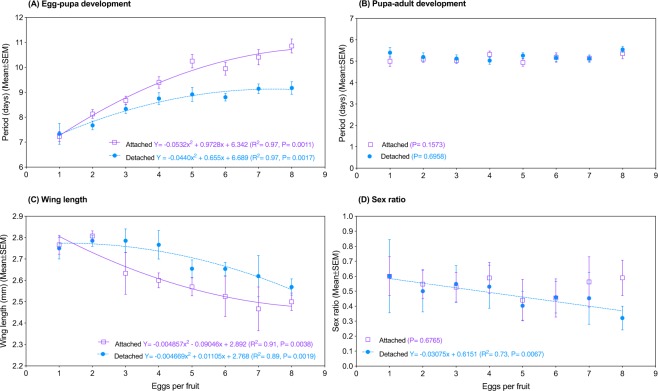
Response of spotted-wing drosophila (*Drosophila suzukii*) to egg density in blueberry fruit when individuals displayed pupation attached to and detached from the fruit. (**A**) Egg-pupa period. (**B**) Pupa-adult period. (**C**) Wing length of females. (**D**) Sex ratio. Curves not shown when P > 0.05. *N* = 19–37.

Attached and detached pupation showed similar egg-pupa periods at 1 egg per fruit, and both sites increased quadratically as egg density rose (attached: *F*_*3,4*_ = 52.53, *P* = 0.0011, R^2^ = 0.97; detached: *F*_*3,4*_ = 42.78, *P* = 0.0017, R^2^ = 0.97, Fig. 4A). Nevertheless, detached pupation supported a much faster development (*F*_*3,10*_ = 33.28, *P* < 0.0001), so much so that at the highest infestation level (8 eggs per fruit) detached larvae became pupae 1.7 day earlier than their attached counterparts (Fig. 4A). No significant effect of egg density was found on pupa-adult period (attached: *F*_*1,6*_ = 2.61, *P* = 0.1573; detached: *F*_*1,6*_ = 0.1684, *P* = 0.6958, Fig. 4B).

### Wing length

At 1 egg per fruit, adults that emerged from attached and detached SWD pupae showed similar wing lengths, and both sites showed a quadratic length decrease as egg density rose (attached: *F*_2,5_ = 20.69, *P* = 0.0038, R^2^ = 0.91; detached: *F*_2,5_ = 28.19, *P* = 0.0019, R^2^ = 0.89, Fig. 4C). However, attached pupation supported stronger length decline (*F*_*3,10*_ = 9.76, *P* = 0.0026).

### Sex ratio

No effect of egg density was observed on the sex ratio of adults that emerged from attached pupae (*F*_1,6_ = 0.1922, *P* = 0.6765). Conversely, in detached pupae, sex ratio linearly decreased as egg density increased (*F*_1,6_ = 16.38, *P* = 0.0067, R^2^ = 0.73, Fig. 4D).

A summary of the effects of larval IC on the 10 biological and behavioral parameters of SWD in blueberry fruit evaluated in this study is shown in Table 1.

**Table 1:** Compilation of the two behavioral and eight biological parameters of the spotted-wing drosophila (*Drosophila suzukii*) addressed in this study, showing the effect of larval intraspecific competition (IC) on each parameter when insects display pupation attached to and detached from a blueberry fruit.

Parameter	IC effect	Attached	Detached	Evidence
**Behavior**				
Pupation site	Yes	−	+	Fig. 2A
Pupation distance	Yes	N/A	+	Fig. 2B,C
**Biology**				
Egg-pupa survival	Yes	N/A	N/A	Fig. 3A
Pupa-adult survival	Yes	−	0	Fig. 3B
Pupae per fruit	Yes	+	++	Fig. 3C
Adults per fruit	Yes	+	++	Fig. 3D
Egg-pupa period	Yes	++	+	Fig. 4A
Pupa-adult period	Yes	0	0	Fig. 4B
Wing length	Yes	−−	−	Fig. 4C
Sex ratio	Yes	0	−	Fig. 4D

“+” = Positive effect (e.g., IC increases the rate of detached pupation).

“−” = Negative effect (e.g., IC decreases the rate of attached pupation).

“0” = Neutral effect (e.g., IC does not affect the pupa-adult period of individuals that were found detached from the fruit).

N/A = Not applicable.

## Discussion

When SWD larvae developed under zero or low levels of IC (2–3.5 larvae/g of host) they preferred to pupate attached to as opposed to detached from the host, in both cornmeal medium and blueberry fruit. This pattern was consistently reversed as IC increased, demonstrating that IC is a key factor for the selection of pupation sites by SWD larvae in both artificial and natural hosts. Observing similar results in the two host types suggests that the IC effect does not necessarily depend on host quality (form, color, texture, nutritional value, etc.). However, it is known that suboptimal nutritional quality can reduce juvenile survival in SWD^29^ thus decreasing IC. Hence, IC will more likely impact pupation behavior in highly nutritious compared to poorly nutritious hosts.

**Figure 5:**
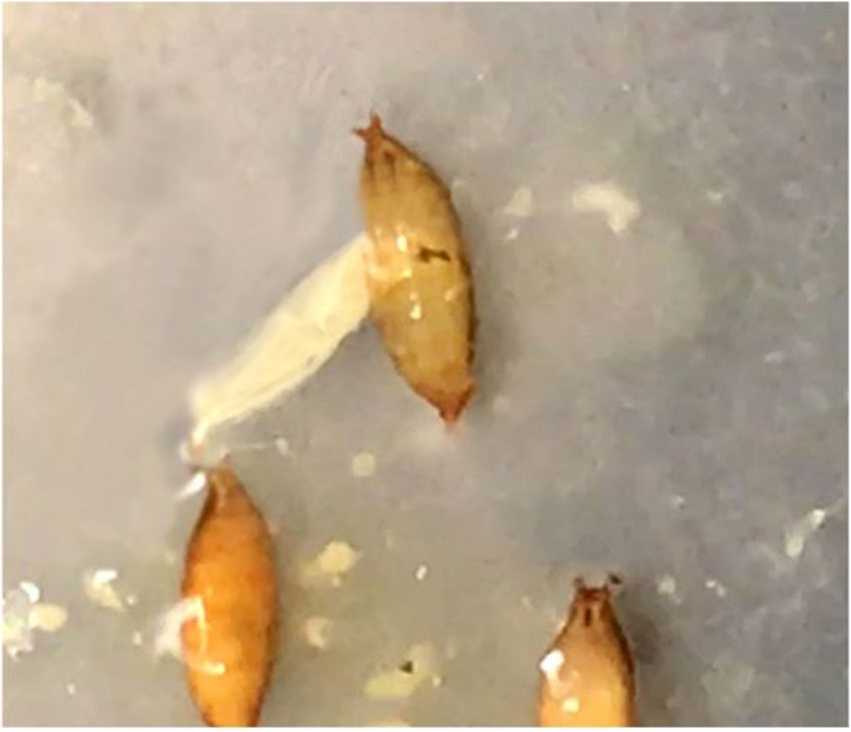
Third instar larva of spotted-wing drosophila (*Drosophila suzukii*) cannibalizing on a conspecific pupa. Note that the anterior part of the larva, including mouthparts, is completely inside the pupa. See Supplementary Video S1 for details of the behavior.

No effect of IC was found on pupation distance in experiments #1 and #2 when 2+ individuals shared the same host. It is important to keep in mind, however, that in both experiments the movement of the wandering larvae was somehow hampered since they couldn’t travel further than the cotton cap. Hence, it is likely that after reaching the cap the larvae wandered up and down inside the tube, randomly pupating in a spot more or less far from the host, likely eliminating any potential effect of IC on pupation distance. On the other hand, the pupation distance of detached SWD larvae was 3x longer under IC relative to no IC, indicating that under absence of competitors little effort is invested in crawling away from the host fruit. In *Drosophila* larvae, crawling is extremely costly in terms of energy^30,31^, and crawling away from the fruit comes with risk of desiccation, predation, parasitism, and exposure to pathogens. Hence, pupation farther away from the host could only be adaptive if the risks of remaining attached to or close to the host were even higher than those of emigrating, e.g., cannibalism and/or drowning. A pupa that is attached to its host has an increased risk of drowning in the host’s contents if immature larvae are still present and actively foraging for food. This phenomenon has been observed repeatedly in our lab colonies when culture plates are highly infested with SWD larvae (Supplementary Fig. S3a). In *Drosophila melanogaster*, the pressure of larval cannibalism under crowded or food-deprived conditions is so high that the species has evolved an anticannibalistic strategy^32–34^. We have observed cannibalized pupae in our SWD colonies, which can be easily spotted because the pupal cases become translucid (empty), feature a hole, and are surrounded by dark circles, probably due to melanization of the hemolymph leaked from the pupae during its consumption by the cannibal larva^35^ (Fig. 5, Supplementary Fig. S3b, Supplementary Video S1). Considering that small larvae tend to cannibalize on large larvae^32,33^, and that sessile individuals such as pupae are especially vulnerable to cannibalism as they cannot physically escape or defend themselves, pupation away from the host can be adaptive for SWD. In fact, this was confirmed when we demonstrated both that SWD larvae that remain within the host are smaller and that attached pupae have lower survival rates as larval density increases. In field conditions, these findings suggest that mature SWD larvae will decrease IC-related mortality by emigrating from a crowded fruit and pupating on twigs or leaves. Additionally, SWD-infested fruit can drop to the soil, where pupae are highly vulnerable to unfavorable environmental conditions and natural enemies^22^. By both shortening their development and displaying detached pupation, as seen in our study, larvae reduce the risk of being inside their host fruit when it drops to the ground.

Interestingly, such shortening had no cost to wing length, an indicator of body size^36,37^. In fact, individuals with a shortened larval period had larger bodies compared to those that emerged from attached pupae. This implies that detached individuals accessed food of higher quality during larval development, likely minimizing the negative IC effects on pupa-adult development and survival relative to attached individuals. In the field, an early-emerging and larger adult will be more fecund^38,39^, as well as will likely have earlier and better access to critical resources such as food, mates, and hosts. The exact mechanism leading to shorter development period and larger size (e.g., increased foraging rate) still remain to be investigated, but our findings clearly show that such a mechanism depends on larval density. It is worthwhile to check if the behavioral and biological patterns found in our study will also be observed in the field. If that happens, control tactics aimed at pupae located in host plants as opposed to soil surface should be developed.

Both the maximum of 2 attached pupae per fruit and the increasing detached pupation as density rose indicate that each SWD larva tolerated only one other larva per fruit, leaving to pupate elsewhere at higher densities. As previously mentioned, the two individuals that remained attached to the fruit were less robust (smaller size) thus were at higher risk of desiccation than their emigrating competitors. Hence, remaining attached to the fruit likely increased their survival chances. In addition, undernourishment may explain why we never found a striking contrast between attached and detached pupation in blueberry (experiment #2) as we did in the cornmeal medium (experiment #1). In the latter case, a considerable amount of medium was still available by the time that larvae selected sites to pupate, meaning that they were well nourished thus more capable of emigrating from the host and less likely to suffer from desiccation.

Besides the effects previously mentioned, IC directly affected several other life history traits of SWD. Particularly interesting results were seen in egg-pupa survival because lower levels of competition (2–3 eggs per fruit) led to higher survival than no competition at all (1 egg per fruit), which characterizes an Allee effect^40^. The ecological mechanism underlying such phenomenon must still be investigated, but habitat alteration by means of inoculation of yeasts or other symbiont microorganisms into the host fruit by the mother during oviposition may play a role^41–43^. Symbiotic relationships between *Drosophila* – including SWD – and yeasts are well documented^44^. Inoculating media with yeast increases pupal production^45^, while its absence decreases egg-pupa survival even at low larval densities^29^. Extra yeast inocula are introduced with every new oviposition. Hence, the more eggs are laid in a fruit, the more the fruit will be inoculated with yeasts, which can serve as a source of nutrients for foraging larvae. Additionally, we do not exclude the capability of yeasts to outcompete harmful microbes^45^. For example, we noticed that blueberry fruits infested with 1 SWD egg were infected with an unidentified post-harvest fungus more frequently than any other egg density, indicating that the fungal infection was kept under control when 2 or more eggs were laid in a fruit. In this case, yeasts could have acted as antagonistic microrganisms, limiting the fungal infection; or the fungus could have been killed directly by larvae, which were present at higher numbers in fruits infested with 2+ eggs.

Increasing IC resulted in decreasing sex ratio (i.e., decreasing female proportion) for detached pupae. Considering that the pupa-adult survival of these individuals was constant across all larval densities, we conclude that as IC rose males were more likely to display detached pupation than females, thus explaining the decreasing sex ratio observed at this pupation site. Because male emigration rates followed IC rates, we expected a sex ratio increment in individuals that remained attached to the fruit, which did not happen. By looking into the pupa-adult survival of attached individuals, we found that such rate linearly decreased as IC increased. This means that as IC rose, the pupae of females died at a higher rate than the pupae of males, thus explaining the constant sex ratio observed in attached insects. We conclude that (1) most of the IC-triggered emigration is formed by males, and (2) males have a survival advantage over females in highly competitive environments. Such survival superiority is possibly an unexpected effect of male smaller size^45,46^, which allow males to require fewer resources than females to complete development and emerge as adults. As to why males are more prone to leave a fruit due to IC than females, we have no explanation.

The ability of SWD to pupate attached or detached from its host fruit has been known for decades^21^, but data about site preferences are just starting to emerge^22^, and the factors that influence such preferences have not yet been fully explored. This study shows that IC among larvae of SWD not only consistently results in detached pupation in both natural and artificial diets, but also stimulates larvae to crawl longer distances from the host fruit before pupating. Additionally, it demonstrates that detached pupation is associated to survival and developmental benefits to SWD individuals, increasing their fitness. Future research should focus on the mechanisms underlying these physiological and behavioral effects, as well as on testing if the patterns found here in this laboratory study are also observed in the field.

## Materials and Methods

### SWD colony

A colony of SWD has been maintained in our laboratory since 2009 from adults and pupae provided by the Horticultural Crops Research Laboratory (USDA-ARS, Corvallis, OR, USA). This colony has been periodically supplemented with wild-caught adults and pupae from the Willamette Valley and Columbia River Gorge of Oregon. About 300–500 SWD adults were kept per Bugdorm mesh cage (30 × 30 × 30 cm) and provided with a sponge soaked in deionized water, as well as a Petri dish (9 × 1.5 cm) containing standard cornmeal medium sprinkled with brewer’s yeast for feeding and oviposition. Dishes containing newly laid eggs were transferred daily to pint plastic freezer containers (8.5 × 8.5 × 5 cm). The Petri dishes remained uncovered, allowing larvae to wander freely within the limits of the pint container. Pint containers were covered with a screened lid to allow air exchange and prevent larval escape. Both adults and juveniles were kept continuously in a climate-controlled chamber (24.1 ± 0.4 °C, 62 ± 8% R.H., and 14:10 L:D photoperiod).

### Experiment 1 – Artificial host

Deionized water was added to Petri dishes containing third-instar (6–7 days old) SWD larvae from the colony to motivate juveniles to emerge to the surface of the diet. Then, larvae were selected at random and carefully transferred to plastic tubes (2 × 9 cm) each containing 3 mL of fresh cornmeal medium, at low, medium or high larval densities (10, 30, or 100 larvae per tube, respectively). Each tube constituted a replicate (*N* = 5–6 per infestation level). All tubes were capped with a cotton plug and kept in the climatic chamber (24.1 ± 0.4 °C, 62 ± 8% R.H., and 14:10 L:D photoperiod). Four days later, each tube was evaluated for 1) number of pupae in the diet versus on the tube wall, 2) survival rate of pupae (pupae/starting larval numbers), and 3) distance between pupa and diet.

### Experiment 2 – Natural host

In order to determine if trends observed from Experiment 1 also can be found under natural field conditions, we tested several levels of realistic field infestation levels on a host, blueberry, that is attacked by this insect. We selected about 40 blueberry fruits (1 ± 0.1 g) were weighed and arranged per Petri dish (9 × 1.5 cm) as shown in Supplementary Fig. S4. Deionized water was added until the point that about half of each berry was covered with water to limit the area available for oviposition, thus facilitating later egg counting. Petri dishes were then introduced into the SWD colonies (Bugdorm cages containing adult flies, see topic “SWD colony” above) for 20, 40, 60 or 120 min, and all eggs laid within fruit were counted under a stereomicroscope. Exposed eggs, i.e., eggs laid on (instead of within) the fruit were removed with a fine, soft brush and eliminated. The exposure periods resulted in infestations of 1, 2, 3…10 + eggs per berry, but only berries carrying 1–8 SWD eggs were used in the study since egg densities higher than that rarely occur under field conditions^27,28^. Each berry constituted a replicate (*N* = 19–37 per infestation level). Following egg counting, fruits were individually placed in 2 × 9 cm plastic tubes capped with a cotton plug and returned to the climate-controlled chamber under the same conditions. Two pieces of filter paper (2 × 7 cm) were introduced in each tube to absorb fruit juices that eventually accumulated at the bottom of the vial due to larval feeding, and to provide shelter to the larvae (simulating leaves and plant crevices). Each tube was checked daily for pupae and emerged adults. The 1) number of pupae, 2) site of pupation (*in*, *on*, or *off* fruit), 3) distance between pupation site and fruit, 4) egg-pupa and 5) pupa-adult periods, 6) number and sex of emerged adults were recorded. The 7) survival rate from egg to pupa (number of pupae/number of eggs), and 8) from pupae to adults (number of adults/number of pupae), 9) secondary sex ratio (number of females/total number of emerged adults), and 10) wing length of females was subsequently determined. Emerged adults were conserved in 70% ethanol. The left wing of each female was carefully extracted under a stereomicroscope with a pair of sharp tweezers (#4a) and fully spread over a drop of deionized water on a glass slide. A ruler was set next to the wing for calibration and the set was photographed with a digital camera coupled either to the stereomicroscope or to a magnifying device (Skyrocket Toys LLC, Los Angeles, CA, USA). The images were subsequently imported to Microsoft^®^ PowerPoint for Mac (v. 15.30) and the length of each wing was measured using the “line” tool (Insert > Shape > Line), from the distal edge of the L3 vein until the proximal edge of the costa proximal vein (Supplementary Fig. S5). The length obtained in the wing measurement was divided by the length of a 1 mm segment in the ruler, resulting in the wing length in mm.

### Statistical analyses

In experiment 1, the number of pupae on the tube wall and in the artificial medium were compared by t-test or Mann-Whitney U-test depending on distributions, with a test at each density. Larva-pupa survival (%) and pupation distance (mm) were compared among the three larval densities by One-Way ANOVA (Kruskal-Wallis followed by Dunn’s method) (Table S1). In experiment 2, One-Way ANOVA (Kruskal-Wallis followed by Dunn’s method) was used to compare pupation *in*, *on*, and *off* fruit (proportion), while Mann-Whitney U test was applied to contrast pupation distance (mm) between larvae under absence and presence of IC. Additionally, polynomial nonlinear regression (1^st^, 2^nd^, or 3^rd^ order) was used to determine whether increasing IC affected any of the different biological parameters under investigation (pupation distance, egg-pupa survival, pupa-adult survival, pupae per fruit, adults per fruit, egg-pupa period, pupa-adult period, wing length, and sex ratio) (Table S1). All curves were fitted to using the least squares (ordinary) fit based on the mean Y value of each point. No constraint or weighting was applied. All analyses were performed using GraphPad Prism version 7.0b for Mac OS X (GraphPad Software, La Jolla, CA, www.graphpad.com).

## Supplementary information

Supplementary Video S1

Supplementary Materials

## Data Availability

The datasets generated during and/or analyzed during the current study are available from the corresponding author upon reasonable request.
