# Supplementary material for: Intraspecific Competition Affects the Pupation Behavior of Spotted-Wing Drosophila (Drosophila suzukii)
Source: Sci Rep. 2019 May 23;9:7775. doi: 10.1038/s41598-019-44248-6 (PMC6533276; doi:10.1038/s41598-019-44248-6)
Supplement: Supplementary file 2 — Supplementary Materials [file 41598_2019_44248_MOESM2_ESM.pdf]

Intraspecific Competition Affects the Pupation Behavior of Spotted-Wing Drosophila (*Drosophila suzukii*)

Cherre S. Bezerra Da Silva[1], Kyoo R. Park[1], Rachel A. Blood[1] & Vaughn M. Walton[1]

[1]Department of Horticulture, Oregon State University, 4017 Agricultural and Life Sciences Building, Corvallis, OR 97331, USA

E-mail: entomologista@gmail.com

Phone: +1 (541) 737 3913

Orcid for CSBDS: 0000-0001-9415-6337

Corresponding author's current address:

Rua Oswaldo Cruz, 1143

Campina Grande, PB, 58428-095, Brazil

Phone: +55 (83) 3182-4300 (extension 4457) | Fax: +55 (83) 3182-4367

Supplementary Materials

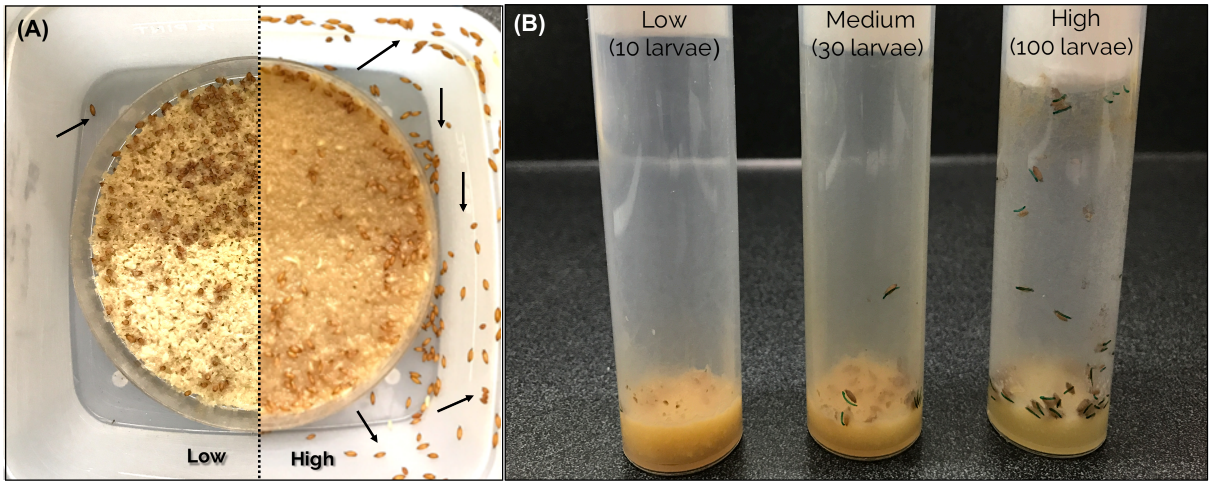

Supplementary Figure S1. (a) Spotted-wing drosophila (SWD, *Drosophila suzukii*) pupal distribution (black arrows) under low and high larval densities in a rearing container of a laboratory colony. (b) SWD pupation sites (cornmeal medium vs. tube wall) under low, medium, and high larval densities (10, 30, and 100 larvae per tube, respectively).

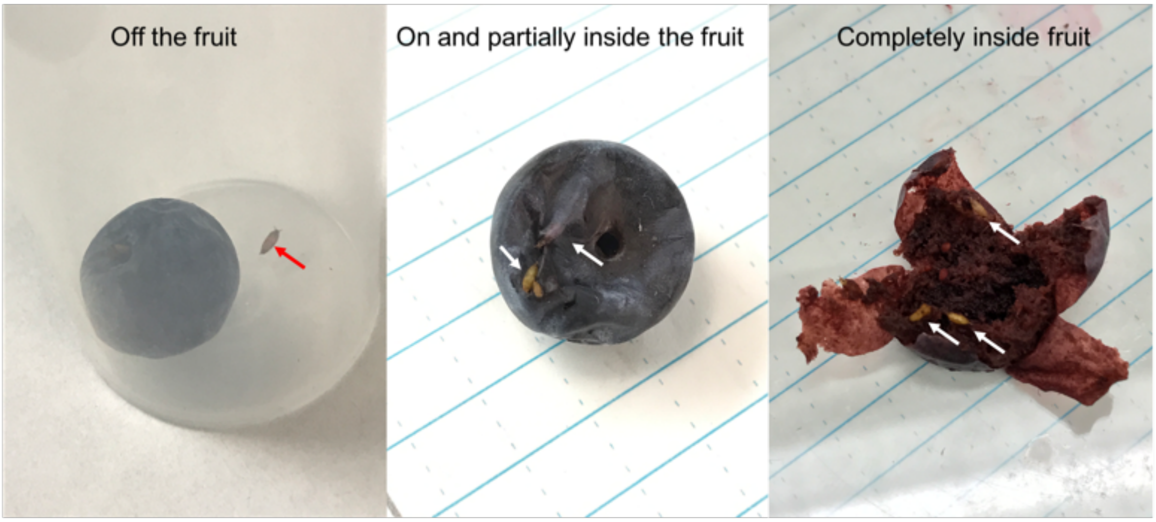

Supplementary Figure S2. Examples of pupation sites selected by mature larvae of spotted-wing drosophila (*Drosophila suzukii*) reared in blueberry in this study. Pupae were found *on*, *off*, as well as partially or completely *in* the fruit.

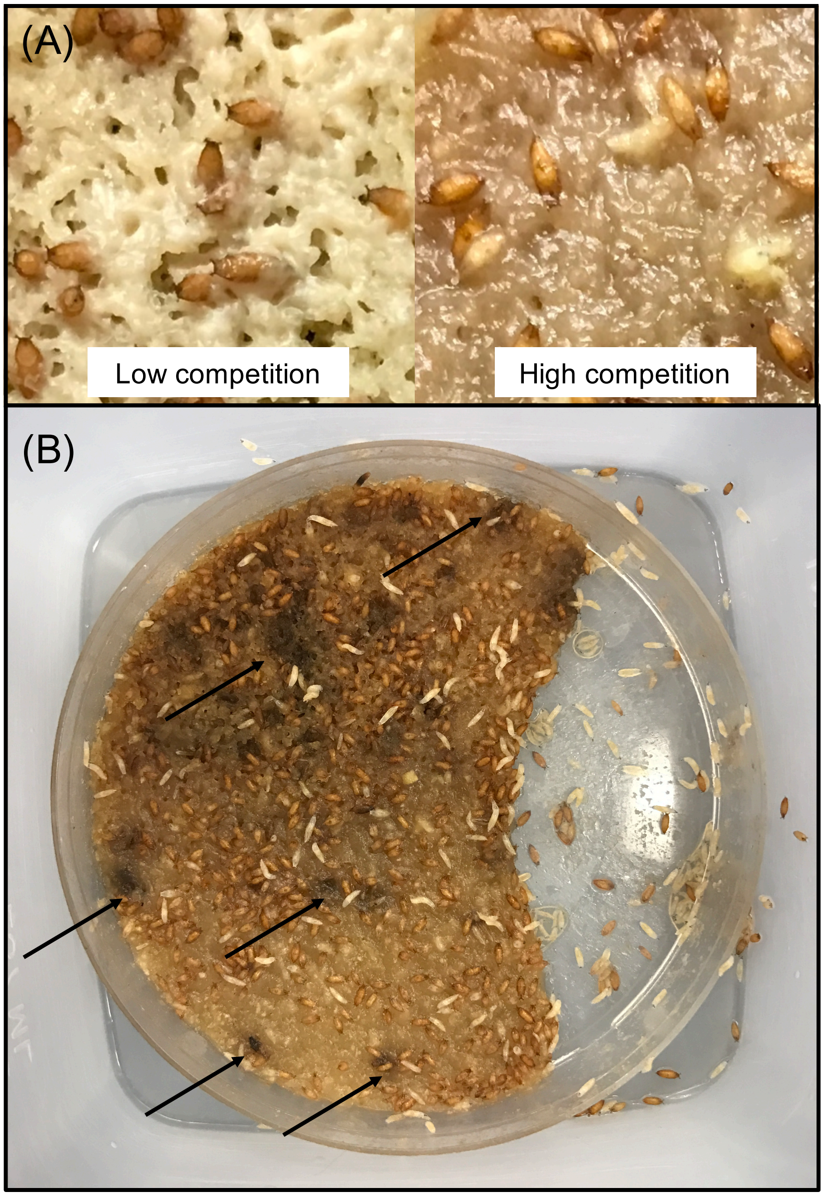

Supplementary Figure S3. (a) At low larval density the pupation of spotted-wing drosophila (SWD, *Drosophila suzukii*) is synchronized and pupae will be found with their anterior part (including breathing openings) pointing upwards, while at high densities pupation is not synchronized and the foraging activity of larvae make pupae to drown within the host. Drowned pupae have their anterior parts covered by the host's contents and die either for lack of air exchange or because such contents block the adults way out from the pupal case. (b) Cannibalized pupae of SWD are circled by dark spots (arrows), which could be formed by melanized hemolymph in the host (cornmeal medium). A feeding hole made by the cannibal larvae is found in the pupal case of cannibalized pupae, evidencing the cannibalism.

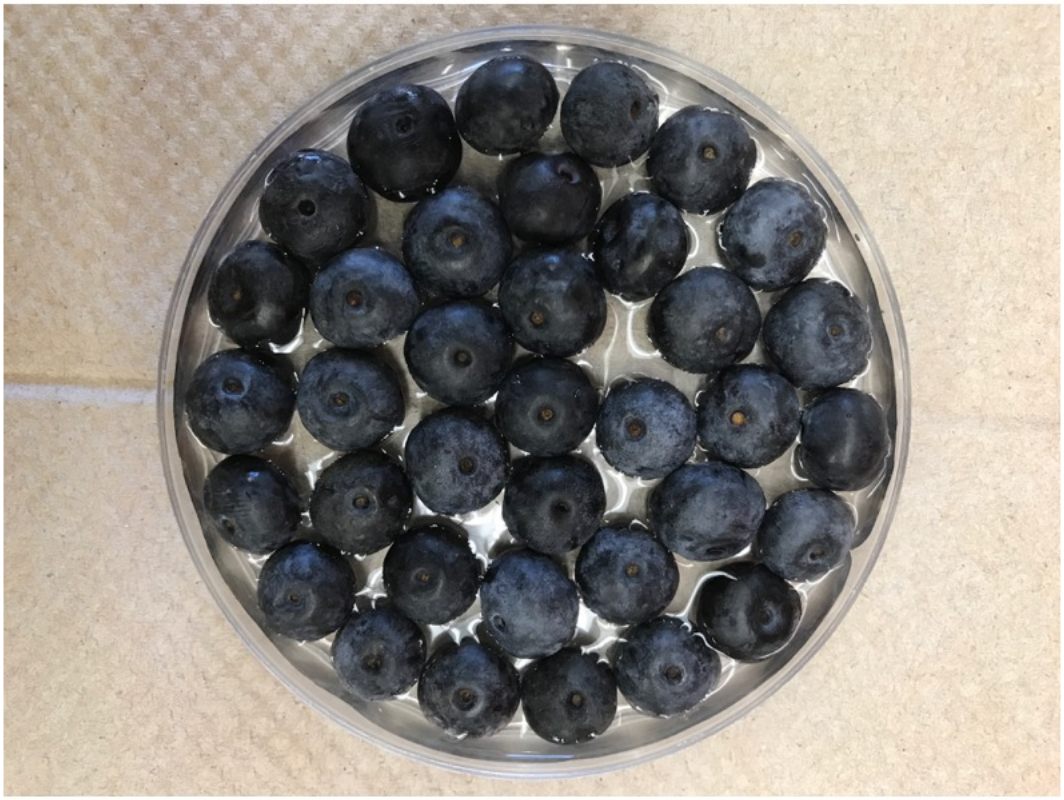

Supplementary Figure S4. Blueberry fruit (1±0.1 g) arrangement in a Petri dish with deionized water to submerge half of each berry. This procedure was performed prior to fruit exposure to gravid females of spotted-wing drosophila (*Drosophila suzukii*) to restrict the oviposition area to the upper portion of the berry, thus enabling more accurate egg counting.

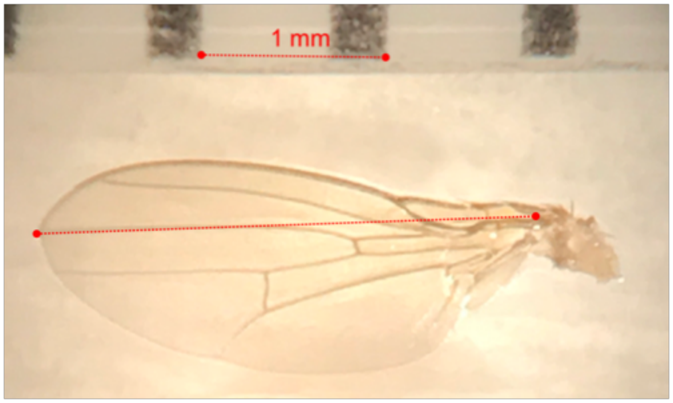

Supplementary Figure S5. Left wing of a female spotted-wing drosophila (*Drosophila suzukii*) showing the two landmarks used to measure its length.

Supplementary Table S1. List of experiments, biological parameters, and respective statistical analysis applied to investigate the effects of intraspecific competition and pupation site on the biology and behavior of spotted-wing drosophila (*Drosophila suzukii*).

Exp.	Effect	Parameter	Test
1	Intraspecific competition (low, medium, and high)	Larva-pupa survival (%)	Kruskall Wallis, Dunn's separation
	Intraspecific competition	Detached pupation (%)	Kruskall Wallis, Dunn's separation
	Intraspecific competition	Pupation distance (mm)	Kruskall Wallis
2	Pupation site (*in* vs. *on* vs. *off* fruit) at 1 egg/berry	Proportion of pupae	Kruskall Wallis
	Intraspecific competition (absence vs. presence)	Pupation distance (mm)	Mann-Whitney U-test
	Intraspecific competition (1-8 eggs per fruit)	Pupation distance (mm)	Polynomial regression
		Egg-pupa survival (proportion)	Polynomial regression
		Pupa-adult survival (proportion)	Polynomial regression
		Number of pupae	Polynomial regression
		Number of adults	Polynomial regression
		Egg-pupa period (days)	Polynomial regression
		Pupa-adult period (days)	Polynomial regression
		Female wing length (mm)	Polynomial regression
		Sex ratio (proportion of females)	Polynomial regression
